# DR1 Activation Inhibits the Proliferation of Vascular Smooth Muscle Cells through Increasing Endogenous H_2_S in Diabetes

**DOI:** 10.14336/AD.2021.1104

**Published:** 2022-06-01

**Authors:** Yuxin Xi, Xin Wen, Yuanzhou Zhang, Lijie Jiao, Shuzhi Bai, Sa Shi, Guiquan Chang, Ren Wu, Fengqi Sun, Jinghui Hao, Hongzhu Li

**Affiliations:** ^1^Department of Pathophysiology, Harbin Medical University, Harbin, Heilongjiang, China.; ^2^School of Medicine, Xiamen University, Xiamen, Fujian, China.; ^3^State Key Laboratory of Oncogenes and Related Genes, Shanghai Cancer Institute, Renji Hospital, Shanghai Jiao Tong University School of Medicine, Shanghai, China

**Keywords:** Dopamine 1-like receptors, vascular smooth muscle cells, H_2_S, diabetes

## Abstract

Tissue ischemia and hypoxia caused by the abnormal proliferation of smooth muscle cells (SMCs) in the diabetic state is an important pathological basis for diabetic microangiopathy. Studies in recent years have shown that the chronic complications of diabetes are related to the decrease of endogenous hydrogen sulfide (H_2_S) in diabetic patients, and it has been proven that H_2_S can inhibit the proliferation of vascular SMCs (VSMCs). Our study showed that the endogenous H_2_S content and the expression of cystathionine gamma-lyase (CSE), which is the key enzyme of H_2_S production, were decreased in arterial SMCs of diabetic mice. The expression of PCNA and Cyclin D1 was increased, and the expression of p21 was decreased in the diabetic state. After administration of dopamine 1-like receptors (DR1) agonist SKF38393 and exogenous H_2_S donor NaHS, the expression of CSE was increased and the change in proliferation-related proteins caused by diabetes was reversed. It was further verified by cell experiments that SKF38393 activated calmodulin (CaM) by increasing the intracellular calcium ([Ca^2+^]_i_) concentration, which activated the CSE/H_2_S pathway, enhancing the H_2_S content in vivo. We also found that SKF38393 and NaHS inhibited insulin-like growth factor-1 (IGF-1)/IGF-1R and heparin-binding EGF-like growth factor (HB-EGF)/EGFR, as well as their downstream PI3K/Akt, JAK2/STAT3 and ERK1/2 pathways. Taken together, our results suggest that DR1 activation up-regulates the CSE/H_2_S system by increasing Ca^2+^-CaM binding, which inhibits the IGF-1/IGF-1R and HB-EGF/EGFR pathways, thereby decreasing their downstream PI3K/Akt, JAK2/STAT3 and ERK1/2 pathways to achieve the effect of inhibiting HG-induced VSMCs proliferation.

Diabetes mellitus is a multifactorial metabolic disorder whose main cause is an absolute or relative lack of insulin, or the peripheral tissues are not sensitive to insulin [1]. Hyperglycemia is a sign of diabetes and is a major risk factor for cardiovascular disease [2]. It has been reported that nearly half of patients with diabetes have at least one type of vascular disease and more than one-third have microvascular disease [3]. The abnormal proliferation of vascular smooth muscle cells (VSMCs) plays a key role in the development of various arterial diseases, including atherosclerosis, arterial restenosis, and hypertension [4]_._ Therefore, preventing the abnormal proliferation of vascular smooth muscle in the diabetic state is a key factor for improving the microvascular complications of diabetic patients.

The physiological actions of dopamine are mediated by five different but closely related G protein-coupled receptors (GPCRs) that are divided into two major groups: D1-like receptors and D2-like receptors [5]. D1-like receptors (DR1) are comprised of D1 and D5 receptors while D2-like receptors (DR2) are comprised of D2, D3 and D4 receptors [5]. The DR1 are generally coupled to Gαs/olf and stimulate the production of the second messenger cyclic adenosine monophosphate (cAMP) and the activity of protein kinase A(PKA) [6]. Meanwhile, DR1 activation increases the concentration of intracellular calcium ([Ca^2+^]_i_). In contrast, DR2 are coupled to Gαi/o and negatively regulate the production of cAMP and the inhibition of PKA, as well as decrease [Ca^2+^]_i_ [6]. Dopamine receptors (DRs) are mostly distributed in the central nervous system, but they are also expressed in peripheral tissues, such as the heart [7], kidney [8], and vascular smooth muscle [9] tissue. The dysregulation of the DRs is a fundamental mechanism of many diseases, including Parkinson’s disease, schizophrenia, hypertension and metabolic dysfunctions or gut motility abnormalities [10]. It has been reported that DR1 can inhibit the hypertrophy, migration and proliferation of VSMCs[11] and reduce the symptoms of atherosclerosis [12]. In the diabetic state, a study of the mechanism of DR1 affecting the proliferation of smooth muscle cells has not been reported yet.

Hydrogen sulfide (H_2_S) has been proven to be a gasotransmitter like carbon monoxide (CO) and nitric oxide (NO). It is endogenously generated and manifests significant effects at physiologically relevant concentrations [13]. The synthesis of H_2_S in mammals is mainly catalyzed from L-cysteine by three key enzymes: 3-mercaptopyruvate sulfurtransferase (3-MST), cystathionine beta-synthase (CBS), and cystathionine gamma-lyase (CSE) [14]. CSE is a key enzyme for endogenous H_2_S production in the cardiovascular system [15]. A large number of studies in recent years have shown that H_2_S regulates the proliferation and apoptosis of many cells, such as cardiomyocytes [16, 17], colon cancer cells [18], and VSMCs [19]. It is worth noting that a lack of endogenous H_2_S is regarded as a risk factor for VSMCs dysfunction [20]. It is particularly important to find substances that can up-regulate endogenous H_2_S to improve VSMCs dysfunction.

Calmodulin (CaM) is a protein that has physiological functions after being activated by binding to calcium ions. It is widely expressed in eukaryotic cells and has a variety of biological meanings [21]. Yang et al. reported that CaM directly interacts with CSE, which activates CSE and thus increases the content of endogenous H_2_S [22]. Interestingly, our previous research found that DR1 had been shown to increase the [Ca^2+^]_i_ in heart tissue and cardiomyocytes [23]. However, it is unclear whether DR1 inhibit the proliferation of VSMCs by regulating the CSE/H_2_S pathway. In general, this study demonstrated that DR1 activates CSE by increasing [Ca^2+^]_i_ and activating CaM, thereby increasing the endogenous H_2_S content and leading to the inhibition of the abnormal proliferation of VSMCs in the diabetic state. Overall, this study opens new paths to the prevention and treatment of diabetes and its complications.

## MATERIALS AND METHOD

### Animal and reagents

C57BL/6J mice were obtained from the Animal Research Institute of Harbin Medical University (Harbin. China). Animals were housed in a climate-and temperature-controlled room on a 12:12-h light-dark cycle. The mice were maintained on a standard diet and water ad libitum. All animal experiments were performed according to the Guide for the Care and Use of Laboratory Animals published by the China National Institutes of Health and approved by the Animal Care Committees of Harbin Medical University (Harbin, China).

SKF38393 (an agonist of dopamine 1 receptors, DR1), PPG (an inhibitor of cystathionine gamma-lyase, CSE), streptozocin (STZ), Sodium hydrogen sulfide (NaHS), AG1024 (an inhibitor of IGF-1), AG490 (an inhibitor of JAK2), PD98059 (an inhibitor of ERK1/2), LY294002 (an inhibitor of PI3K), Tetracaine (a RyR inhibitor), 2-APB (an inhibitor of IP_3_) and AG1478 (an inhibitor of EGFR) were purchased from Sigma Chemical Co. (St. Louis, MO, USA). The primary antibodies for CSE, Cyclin D1, PCNA, P21 and β-actin were from Proteintech (Wuhan, China). The 7-azido-4-methyl-coumarin (H_2_S probe), CaM, t-ERK1/2, p-ERK1/2, t-PI3K, p-PI3K, t-Akt, p-Akt, t-JAK2, p-JAK2, t-STAT3, p-STAT3, HB-EGF, IGF-1, t-EGFR, p-EGFR, t-IGF-1R and p-IGF-1R antibodies were obtained from Cell Signaling Technology (Denver, CO, USA). DR1 antibody was purchased from GENE TEX (California, USA). The protein A/G Magnetic Beads was obtained Selleckchem, (Houston, TX, USA). The Cell Counting Kit-8 (CCK-8) was obtained from Boster Bio-engineering Limited Company (Wuhan, China). The EdU was obtained from RiboBio Company (Guangzhou, China). Control vector plasmid and over-DR1 plasmid, control siRNA, CSE siRNA, IGF-1 siRNA, HB-EGF siRNA and calcein AM were purchased from Santa Cruz (Bergheimer, Germany). All other chemicals were from Sigma or Santa Cruz.

### Diabetes model and treatment protocols

**Figure 1.**
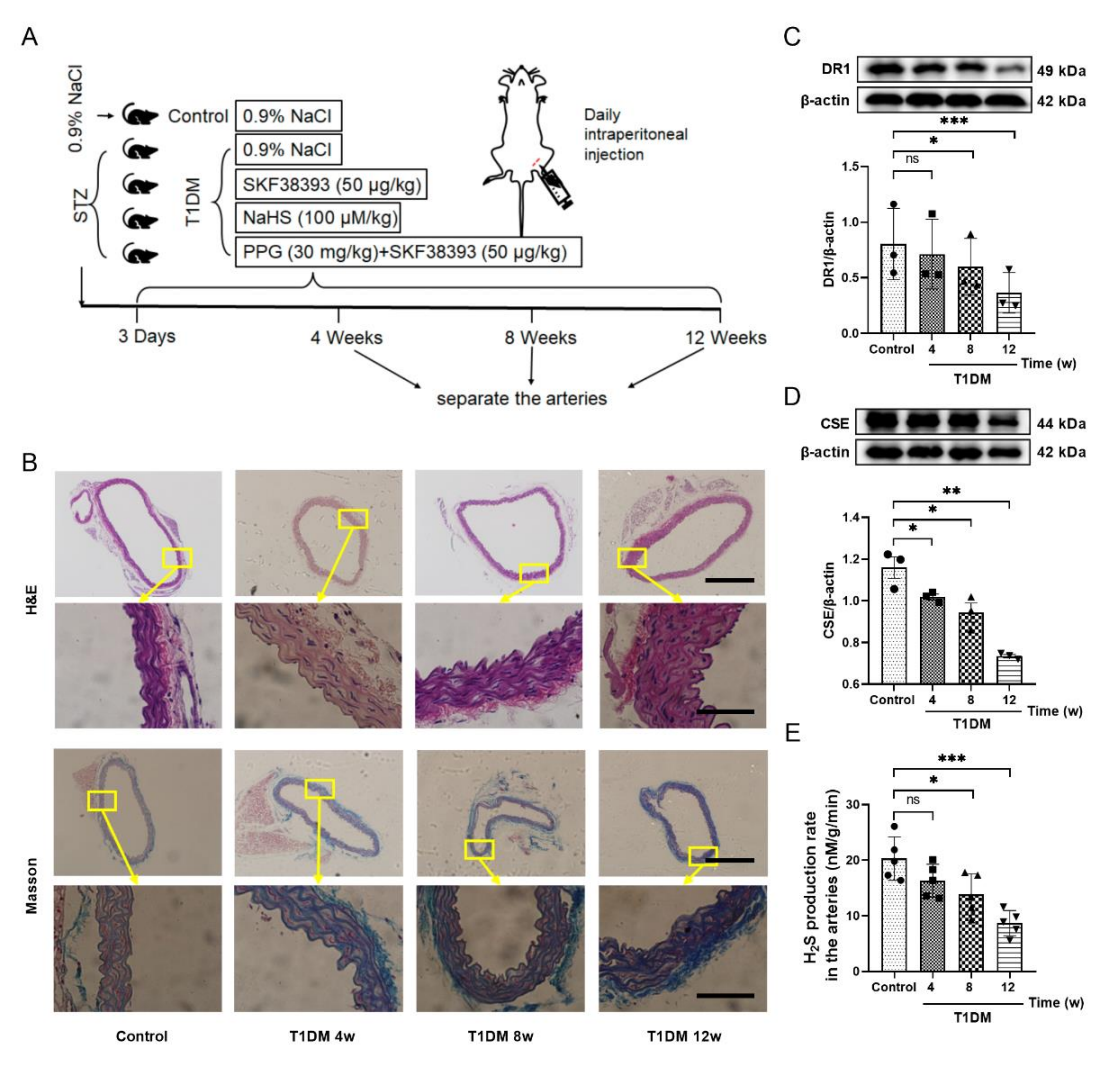
Characteristics of the aorta in diabetic mice and changes in the expression of DR1 and CSE and hydrogen sulfide content in diabetic mice. (A) Schematic diagram of animal experiment grouping. (B) H&E and Masson staining results of control and 4w, 8w, 12w diabetic mice aorta (100 × or 600 × magnification). Scale bar in aortic ring: 500 μm; Scale bar in part of aorta: 100 μm. (C and D) Western blot analysis showed the expression of DR1 and CSE of the arteries in different time points of T1DM mice. The intensity of each band was quantified by densitometry, and data were normalized to the β-actin signal. (E) The production of H_2_S in the arteries of different time points of T1DM mice. All data were from at least 3 independent experiments. The results were expressed as the mean±SEM. *p < 0.05, **p < 0.01, ***p < 0.001 vs. control group. ns, not significant.

After animal adaptation to the environment for 1 week. Both male and female C57BL/6J (6-weeks-old, 20-22g) mice were made diabetes by a single injection of streptozotocin (150 mg/kg, intraperitoneally). The STZ was dissolved in 100 μM citrate buffer (citric acid: sodium citrate=1:1.32). After 3 days, the mice with glucose levels above 16.67 mmol/L were considered hyperglycemic (diabetes) [24]. Simultaneously recorded the daily water intake and food intake of these diabetic mice and monitored the weekly weight changed. At the same time, monitored blood glucose weekly to eliminate mice whose blood glucose had been returned to blow 16.67 mmol/L. Briefly, the experimental treatments were as follows (n=8) (Fig. 1A): (1) Control group (Control): The mice were injected with the same volume of Stroke-physiological saline solution daily. (2) Diabetes group (T1DM): The diabetic mice were injected with the same volume of Stroke-physiological saline solution daily. (3) Diabetes+SKF38393 group (T1DM+SKF38393): The diabetic mice were injected with SKF38393 (50 μg/kg) daily. (4) Diabetes+NaHS group (T1DM+NaHS): The diabetic mice were injected with NaHS (100 μM/kg) daily. (5) Diabetes+PPG+SKF38393 group (T1DM+ PPG+SKF38393): The diabetic mice were simultaneously injected with SKF38393 (50 μg/kg) and PPG (30 mg/kg) daily. All drugs were dissolved in Stroke-physiological saline solution, and all the above mice were injected intraperitoneally. After the treatment reached 4, 8 and 12 weeks, the mice aorta samples were obtained. Fix the samples in glutaraldehyde for electron microscopy Studies and 4% paraformaldehyde for H&E, Masson and immunohistochemical staining.

### Aorta H&E and Masson staining

Aorta tissue samples were fixed in 10% neutral buffered formaldehyde and dehydrated through a serial alcohol gradient and embedded in paraffin wax blocks. 4-um-thick aorta tissue sections were dewaxed in xylene, rehydrated through decreasing concentrations of ethanol, and washed. And then stained with H&E for general histopathological evaluation and Masson for show the fibers in the tissue.

### Immunocytochemistry (IHC)

IHC was conducted as described previously [25]. The aorta tissues were fixed in 10% formalin and subsequently embedded in the paraffin. Serial 4 μm-thick sections were prepared. After deparaffinizing and rehydrating, slides were covered with Tris-EDTA (pH 9.0) buffer for microwave antigen retrieval. Then, slides were incubated with 3% H_2_O_2_ solution for 10 minutes. The sections were blocked for 1 hour with 5% goat serum and incubated with α-SMA primary antibodies overnight at 4 °C. Next, the slides were incubated with the secondary antibody for 30 minutes at 37? following the instruction. For signal detection, the slides were incubated with DAB solution for 3 minutes. Finally, slides were counterstained with hematoxylin. All images were acquired by microscope with 10× or 60× objectives.

### Culture of MOVAS

The mouse aortic SMCs line (MOVAS)were purchased from the Shanghai Institute of Biochemistry and Cell Biology (Shanghai, People's Republic of China) and were cultured in growth medium DMEM containing 10% foetal bovine serum (FBS), 100 U/ml penicillin, and 100 mg/ml streptomycin. The experiments were performed when the cells reached 70-80% confluence by trypsinization according to the standard procedures and the medium was changed every 48 h.

### Experimental groups of cell model

The MOVAS were randomly divided into the following 7 groups and each group included 6 samples (n=6): (1) Control group (Control): The MOVAS were cultured 2% FBS-DMEM containing 5.5 mM glucose; (2) High glucose group (HG): The MOVAS were cultured with 2% FBS-DMEM containing HG (40.0 mM glucose); (3) HG+SKF38393 group: 5 μM SKF38393 was added to the medium containing HG at the same time; (4) HG+NaHS group: 100 μM NaHS was added to the medium containing HG at the same time; (5) HG+PPG+SKF38393 group: 5 mM PPG was added to the medium for 1h before HG and SKF38393 incubation, the other procedure was similar to that for group 3; (6) HG+2-APB (or W-7 or Tetracaine)+SKF38393 group: The procedure was similar to that for group 5, except that PPG was replaced by 3 μM 2-APB (or 10 μM W-7 or 100 μM Tetracaine); (7) HG+AG1024 (or AG1478, or PD98059, or LY294002) group: 5 μM AG1024 (or 100 nM AG1478, or 10 μM PD98059, or 10 μM LY294002) was added to the medium for 1h before HG incubation. Then all the MOVAS were incubated for 48 h.

### MOVAS proliferation assay

Cells were seeded in 96-well plates (3 × 10^3^ cells/well). After the cells were serum-starved for 12 h, exposed to each treatment for 48 h. The cell counting kit-8 (CCK-8) was added to each well according to the manufacturer's introductions. Optical density was measured by a microplate spectrophotometer at a wavelength of 570 nm (A570).

We used the same processing method to detect cell Edu proliferation. Detection of EdU was achieved with the Cell-Light EdU Apollo 488, according to the manufacturer’s protocol. Images were captured by a Fluorescence microscope.

### Detection of H_2_S production rate in arteries

H_2_S production rate was measured as described previously [14]. In brief, after different treatments, the arterial tissue was collected and homogenized in 50 mM ice-cold potassium phosphate buffer (pH 6.8). The flasks containing the reaction mixture (100 mM potassium phosphate buffer, 10 mM L-cysteine, 2 mM pyridoxal 5-phosphate, and 10% cell homogenates) and center wells containing 0.5 mL 1% zinc acetate and a piece of filter paper (2 × 2.5 cm) were flushed with N_2_ gas and incubated at 37°C for 90 min. The reaction was stopped by adding 0.5 mL of 50% trichloroacetic acid, and the flasks were incubated at 37°C for another 60 min. The contents of the center wells were transferred to test tubes, each containing 3.5 mL of water. Then 0.5 mL of 20 mM, *N*-dimethyl-*p*-phenylenediamine sulfate in 7.2 M HCl and 0.5 mL 30 mM FeCl_3_ in 1.2 M HCl was added. The absorbance of the resulting solution at 670 nm was measured 20 min later with a FLUO star OPTIMA microplate spectrophotometer.

### Analysis of H_2_S production rate in HG-induced MOVAS

The fluorescence intensity of H_2_S in the MOVAs was tested by using 7-azido-4-methylcoumarin(C-7Az), which has been proved to selectively respond to H_2_S as described previously [26]. MOVAS cells were seeded at an equal number of cells (2.0×10^5^ per plate) in 60 mm^2^ plates in the medium containing 10% FBS. After 48 hours of incubation, the plates were washed with PBS three times and then incubated with PBS containing 50 μM C-7Az for 30 min at 37?, followed by washing of the cells with PBS for three times. Images were captured by a fluorescence microscope and fluorescence enzyme-labeled instrument.

### Western blot

The related protein expressions were measured by Western blot as described previously [27]. Briefly, whole cells or arterial tissues were lysed using RIPA lysis buffer with 10% protease inhibitor for 30min. After 12000?g centrifugation for 30?min, the protein concentration of the extracts was quantified using BCA kit and protein samples were separated by SDS-PAGE and transferred onto PVDF membranes. The membranes were blocked in 5% non-fat milk for 1h, then incubated with primary antibody overnight at 4 °C. Then the membrane was incubated with horseradish peroxidase-conjugated secondary antibodies at room temperature for 1h. The membrane was washed three times with TBS-T for 10 min Before and after this step. The protein bands were detected using enhanced chemiluminescence (ECL) reagent, and identified and quantified using Image J.

### Overexpression and siRNA transfection

Transfection of MOVAS cells by over-DR1 and control vector or CSE siRNA, IGF siRNA, EGFR siRNA and corresponding control siRNA were achieved by using the Lipofectamine™ 3000 transfection agent from Invitrogen (Burlington, ON) as described previously [17]. In brief, the MOVAS were seeded at equal number of cells (2.0×10^5^ per plate) in 60 mm^2^ plates with the medium containing 10% FBS. The cells were plated to form 60-70% confluent monolayers for siRNA transfection, and 80-90% confluence for plasmid transfection. siRNA or plasmid and the transfection reagent complex were added to the serum-free medium for 4 h, and the transfection continued for another 48 h (for siRNA transfection) or 24 h (for plasmid transfection) in serum-containing regular medium. After that, the cells were collected for detection of protein expressions with western blotting analysis.

### Determination of intracellular calcium concentration

Intracellular calcium concentration ([Ca^2+^]_i_) was measured by Ca^2+^ indicator Fluo 4-AM as described previously [28]. MOVAS cells were seeded in light-proof 96-well plates (3 × 10^3^ cells/well) in the medium containing 10% FBS. After 48 h of each treatment, the Fluo 4-AM was added to each well according to the manufacturer's introductions. In detail, cells were incubated with Fluo-4 AM (final concentration of 0.5 uM) for 30 min in PBS at 37? then washed three times with PBS and incubated for an additional 20 min to ensure that Fluo-4 AM is completely converted in the cells. Images were captured by a fluorescence microscope and fluorescence enzyme-labeled instrument.

### Co-immunoprecipitation

The interaction of DR1 and CSE was detected via Co-immunoprecipitation as described previously [22]. MOVAS cells were seeded at an equal number of cells (2.0×10^5^ per plate) in 60 mm^2^ plates in the medium containing 10% FBS. After treatments, cells were lysed using RIPA lysis buffer with 10% protease inhibitor for 30min. After 12000?g centrifugation for 30?min, the lysates were immunoprecipitated with 2?μg CSE or CaM antibody overnight at 4?°C before coupled to Protein A/G Magnetic Beads for 2?h according to the instructions of Protein A/G Magnetic Beads for IP. After that, western blotting was performed using the above methods of immunoblotting.

### Statistical analysis

All data are expressed as the mean±SEM and represent at least three independent experiments. Statistical comparisons were made using Student’s *t*-test or one-way ANOVA followed by a post hoc analysis (Tukey test) where applicable. The significance level was set at p<0.05.

## RESULTS

### Characteristics of the aorta in diabetic mice

In order to simulate the proliferation of VSMCs in the diabetic state, streptozocin (STZ) was used to induce C57BL/6J mice to become a type 1 diabetes mellitus (T1DM) model. The aorta of the mice was stained with α-SMA, and it was found that it was strongly stained brown, which proved that the aorta of the mice was mainly comprised of SMCs (Supplementary Fig. 1). The blood glucose of diabetic mice was always maintained above 16.7 mmol/L. Compared with the control group of mice, their blood glucose, water intake and food intake increased, which is consistent with the symptoms of diabetes (Supplementary Tab. 1). By observing the results of aortic staining, it was found that the thickness of the media in the aorta of the diabetic mice increased, and the accumulation of collagen in the media increased as the duration of diabetes increased (Fig. 1B). This phenomenon shows that the diabetic condition of the mice is aggravated. Compared with the control group, the expression of DR1 and CSE and the endogenous H_2_S content of the mice arteries also showed a downward trend with the progression of the disease, and it reached its lowest level at 12 weeks (Fig. 1C, D and E). Thus, 12-week diabetic mice were used in the follow-up experiments. The data of supplementary Tab. S1 showed that the blood glucose, water intake and food intake were not different between control 4 and 8 as well as 12 weeks. In order to correspond with the 12-week diabetic mice, we selected control 12 weeks as control group in the follow-up experiments.

**Figure 2.**
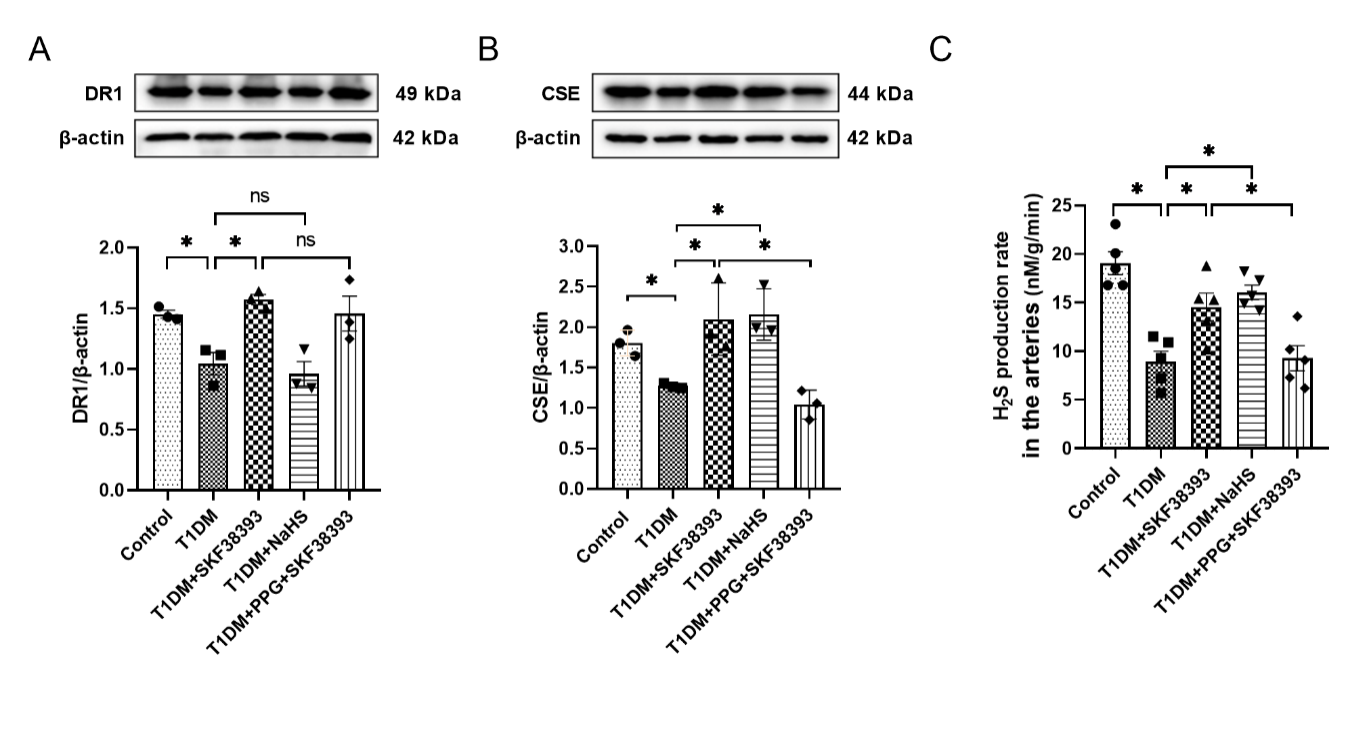
SKF38393 and NaHS affect the expression of DR1, CSE and H_2_S production in the arteries of T1DM mice. The expression of DR1 (A) and CSE (B) was detected by Western blot in the arteries of T1DM mice. The intensity of each band was quantified by densitometry, and data were normalized to the β-actin signal. (C) H_2_S production of the arteries in T1DM mice. The experiments were repeated at least three times. The results were expressed as the mean ± SEM. Significant differences are indicated as * p < 0.05. ns, not significant.

### DR1 activation increases the production of endogenous H_2_S in the arteries from T1DM mice

Compared with the control group, the expression of DR1 and CSE and the production rate of endogenous H_2_S were decreased in the T1DM group. Compared with the T1DM, DR1 agonist SKF38393 significantly increased DR1 and CSE expression and endogenous H_2_S production, while NaHS (a H_2_S donor) only increased CSE expression and endogenous H_2_S production and had no effect on DR1 expression. The CSE inhibitor PPG abolished the effect of SKF38393 (Fig. 2).

### DR1 activation inhibits VSMCs proliferation by increasing endogenous H_2_S in T1DM mice

**Figure 3.**
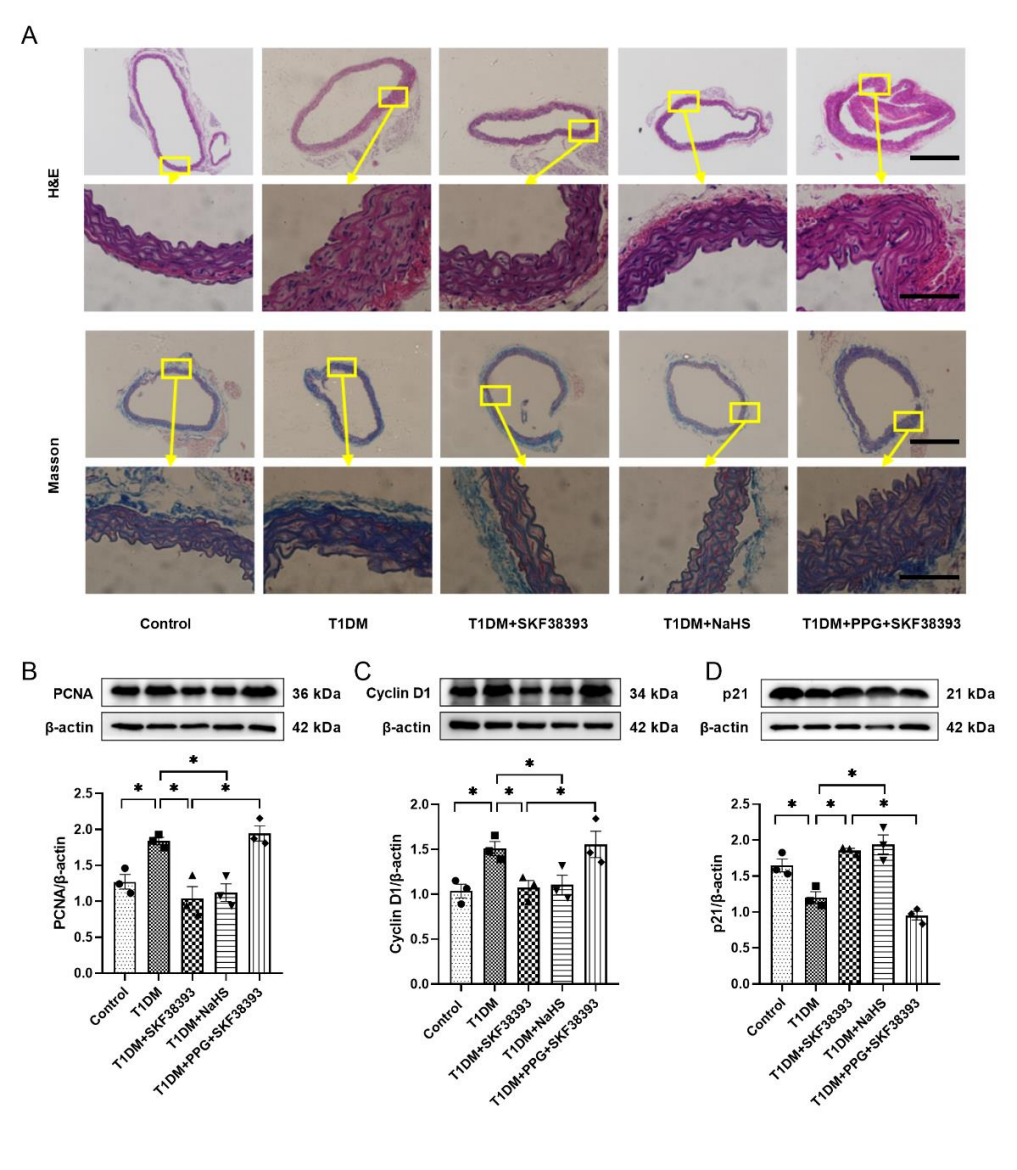
SKF38393 and NaHS inhibit VSMCs proliferation in T1DM mice. (A) H&E and Masson staining results of the aorta from different experimental groups in T1DM mice (100 × or 600 × magnification). Scale bar in aortic ring: 500 μm; Scale bar in part of aorta: 100 μm. The expression of PCNA (B), Cyclin D1 (C) and p21 (D) was tasted by Western blot in the arteries of T1DM mice. The intensity of each band was quantified by densitometry, and data were normalized to the β-actin signal. The experiments were repeated at least three times. The results were expressed as the mean ± SEM. Significant differences are indicated as * p < 0.05.

As shown in Figure 3, the aortic SMCs proliferation, collagen deposition and the expression of PCNA and Cyclin D1 were increased and the expression of p21 was decreased in the T1DM group relative to the control group. The T1DM+SKF38393 and T1DM+NaHS significantly reduced the aortic SMCs proliferation, collagen deposition and PCNA and Cyclin D1 expression and increased p21 expression in comparison with the T1DM group. PPG abolished the beneficial effect of SKF38393.

### DR1 activation increases endogenous H_2_S production in HG-induced MOVAS

**Figure 4.**
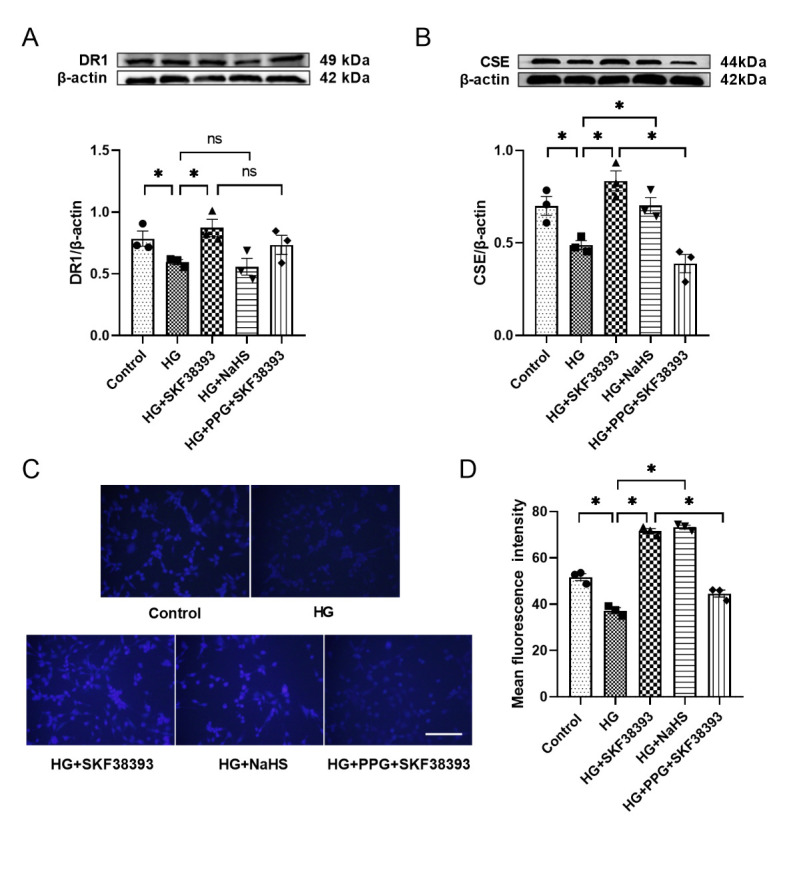
The expression of DR1 and CSE and the production of H_2_S in HG-induced MOVAS. The expression of DR1 (A) and CSE (B) of MOVAS was tested by Western blot. The intensity of each band was quantified by densitometry, and data were normalized to the β-actin signal. (C) The production of H_2_S was observed by fluorescence microscope (200 × magnification). Scale bar: 200?μm. (D) Quantify the results of (C) with a histogram. All data were from three independent experiments. The results were expressed as the mean ± SEM. Significant differences are indicated as * p < 0.05. ns, not significant.

In order to further study the mechanism of DR1 and H_2_S, the mouse aortic smooth muscle cell line MOVAS was used to simulate diabetes in mice. High-dose glucose (HG, 40 mM) simulated diabetic hyperglycemia. We found that the expression of DR1 and CSE and the level of H_2_S was decreased in the HG group (vs. the control group). The addition of SKF38393 increased the above-mentioned indicators (vs. the HG). The addition of NaHS did not increase the expression of DR1 but it increased the expression of CSE and the level of H_2_S (vs. the HG). PPG reversed the up-regulation of SKF38393 on CSE expression and H_2_S production, but did not reverse the up-regulation of DR1 (Fig. 4).

### DR1 activation inhibits cell proliferation by increasing endogenous H_2_S in HG-induced MOVAS

**Figure 5.**
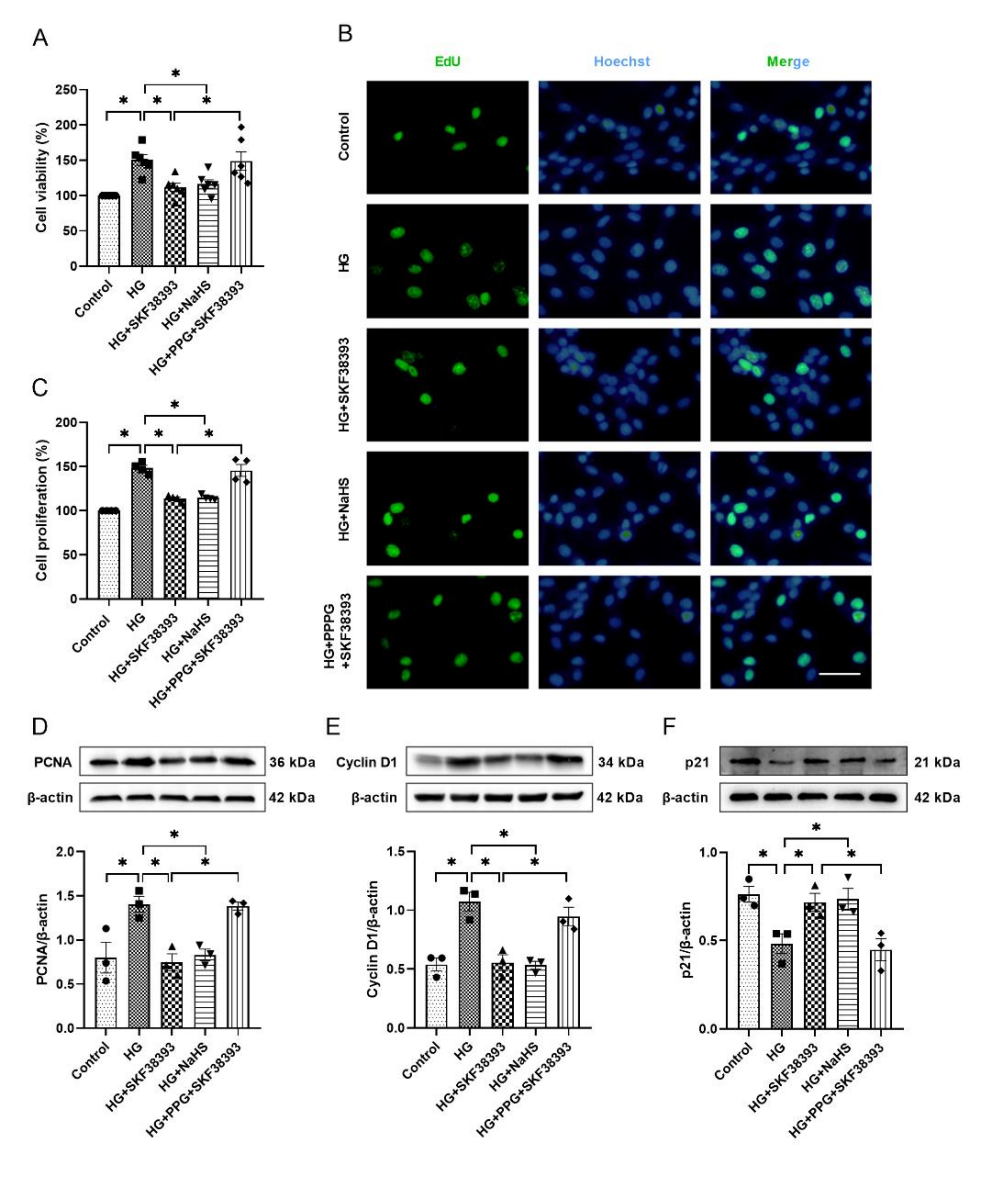
Effects of SKF38393 and NaHS on the proliferation in HG-induced MOVAS. (A) Cell viability was detected by CCK8 kit (n=6). (B) EdU was used to detect cell proliferation by fluorescence microscope (n=4, 200 × magnification). Scale bar: 100 μm. (C) Quantify the results of (B) with a histogram. The expression of PCNA (D), Cyclin D1 (E) and p21 (F) was tasted by Western blot in HG-induced MOVAS (n=3). The intensity of each band was quantified by densitometry, and data were normalized to the β-actin signal. The results were expressed as the mean ± SEM. Significant differences are indicated as * p < 0.05.

The cell viability and proliferation and the expression of PCNA and Cyclin D1 were higher and the expression of p21 was decreased in the HG group than in the control group. SKF38383 and NaHS reversed the effect of HG. PPG abolished the effect of SKF38393 on cell proliferation (Fig. 5).

### DR1-CSE/H2S pathway activation inhibits the proliferation of MOVAS in HG-induced by down-regulating IGF-1/IGF-1R, HB-EGF/EGFR as well as their downstream PI3K/Akt, JAK2/STAT3 and ERK1/2 pathways

**Figure 6.**
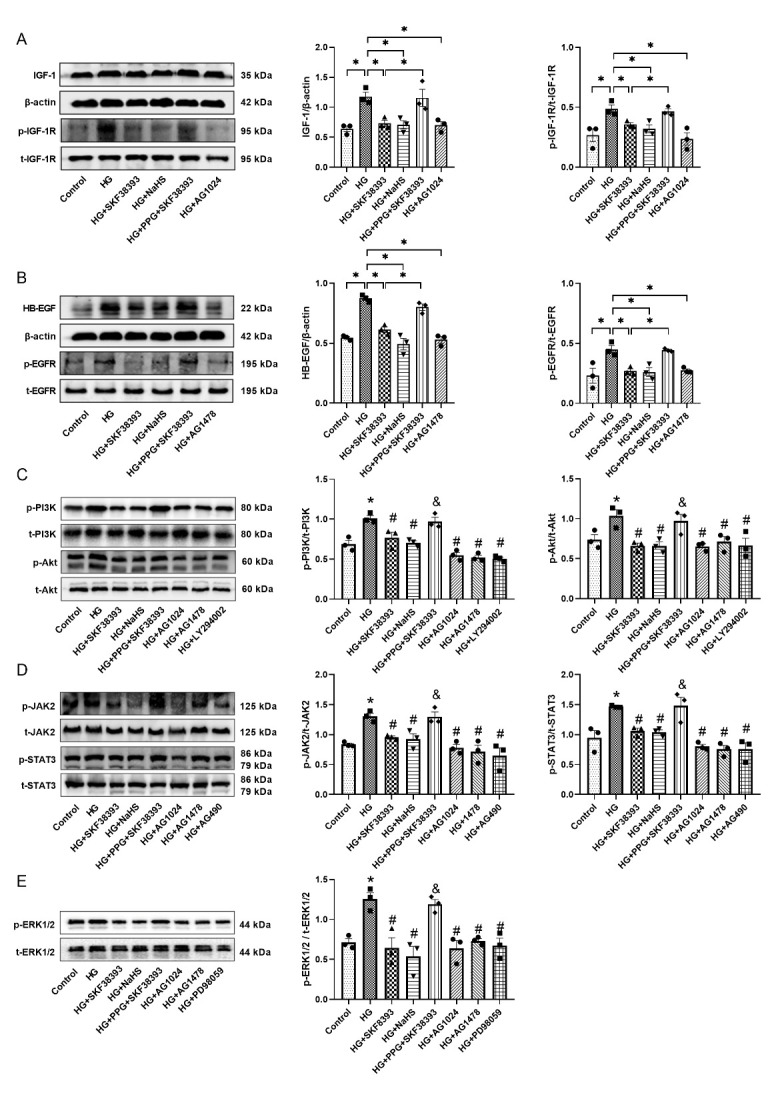
Effects of SKF38393 and NaHS on proliferation-related pathways of HG-induced MOVAS. (A) The expression of IGF-1 and the level of p-IGF-1/t-IGF-1 were detected by Western blot. (B) The expression of HB-EGF and the level of p-EGFR/t-EGFR were detected by Western blot. The expression of p-PI3K/t-PI3K (C), p-Akt/t-Akt (C), p-JAK2/t-JAK2 (D), p-STAT3/p-STAT3 (D) and p-ERK1/2/t-ERK1/2 (E) was detected by Western blot. AG1024 is an IGF-1R inhibitor, AG1478 is an EGFR inhibitor, LY294002 is a PI3K inhibitor, AG490 is a JAK2 inhibitors, PD98059 is an ERK1/2 inhibitor. The intensity of each phosphorylated band was quantified by densitometry, and data were normalized to the corresponding total band signal. All data were from three independent experiments. The results were expressed as the mean ± SEM. Significant differences are indicated as * p < 0.05 which in A and B. * p < 0.05 *vs*. control group; # p <0.05 *vs*. HG group; & p < 0.05 *vs*. HG+SKF38393 group which in C-E.

In order to further verify the mechanism of the DR1-CSE/H_2_S pathway to inhibit proliferation, IGF-1 inhibitor AG1024, HB-EGF inhibitor AG1478, ERK1/2 inhibitor PD98059, PI3K/Akt inhibitor LY294002 and JAK2/STAT3 inhibitor AG490 were used to inhibit the related proliferation pathways. The experimental results showed that in the HG group, the pathway of IGF-1/IGF-1R, HB-EGF/EGFR, PI3K/Akt, JAK2/STAT3 and ERK1/2 was up-regulated compared with the control group. Compared with the HG group, SKF38393 and NaHS markedly down-regulated the above-mentioned pathways. PPG reversed the effect of SKF38393. Moreover, the effect of SKF38393 on the above-mentioned pathways was similar to that of inhibitors of the corresponding pathways (Fig. 6A-E). Meanwhile, both the IGF-1 inhibitor AG1024 and the HB-EGF inhibitor AG1478 suppressed the PI3K/Akt, JAK2/ STAT3 and ERK1/2 pathways (Fig. 6C-E).

**Figure 7.**
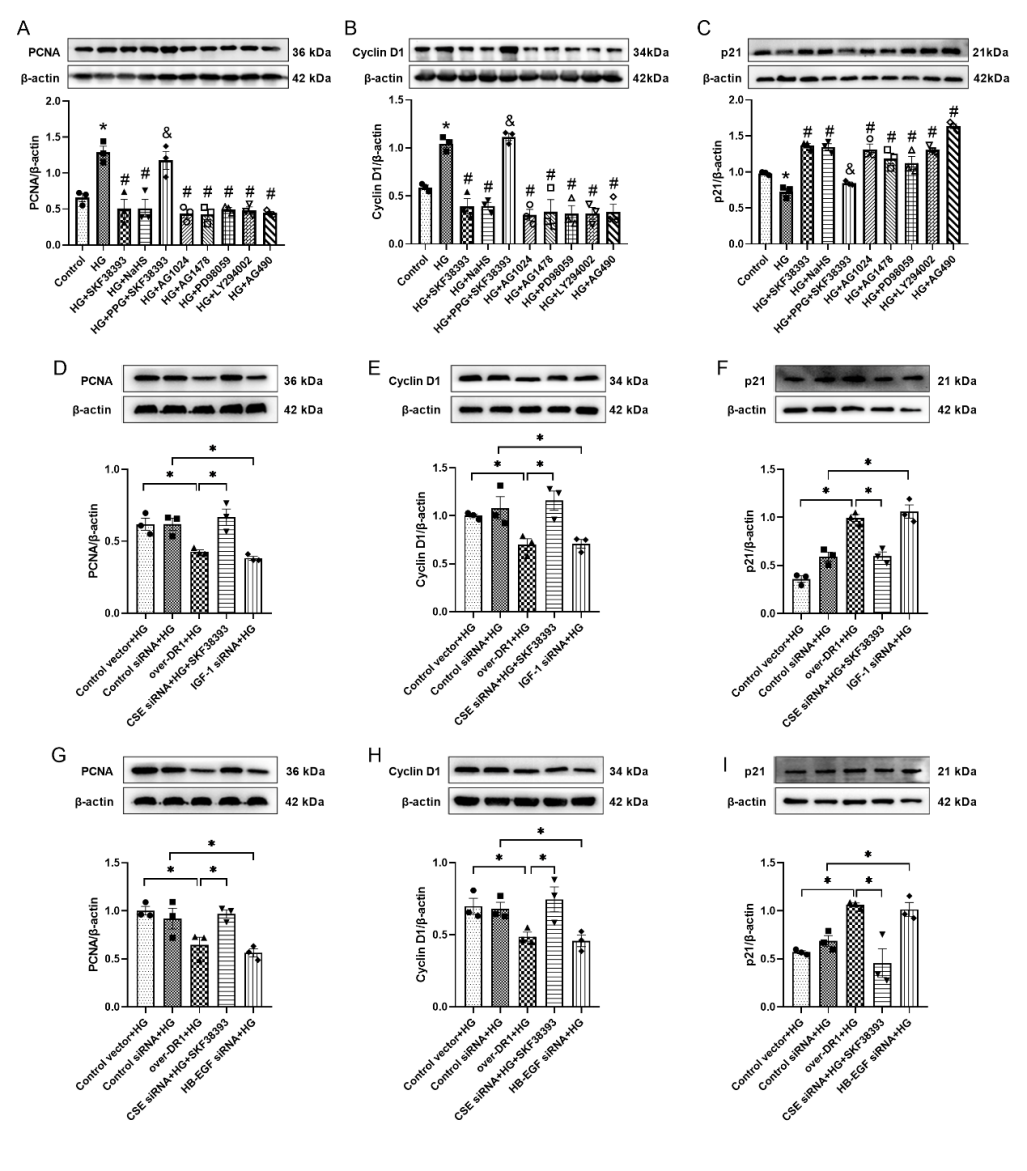
The expression of PCNA, Cyclin D1 and p21 from different treatments in HG-induced MOVAS. Western blot analysis showed the expression of PCNA, Cyclin D1 and p21in different experiment groups. Over-DR1 is the overexpression of DR1 gene. CSE siRNA is knock down CSE gene. IGF-1 siRNA is knock out IGF-1 gene. HB-EGF siRNA is knock down HB-EGF gene. The intensity of each band was quantified by densitometry, and data were normalized to the β-actin signal. All data were from three independent experiments. The results were expressed as the mean ± SEM. * p < 0.05 *vs*. control group; # p <0.05 *vs*. HG group; & p < 0.05 *vs*. HG+SKF38393 group which in A-C. Significant differences are indicated as * p < 0.05 which in D-I.

In addition, we detected the expression of proliferation-related proteins (PCNA, Cyclin D1 and p21) by treatment with their respective pathway inhibitors. Our data showed that the expression of PCNA and Cyclin D1 were higher and the expression of p21 was decreased in the HG group relative to the control group. SKF38383 and NaHS reversed the effect of HG. PPG completely abolished the beneficial effect of SKF38393. The effect of SKF38393 on the expression of proliferation-related proteins was similar to that of AG1024, AG1478, PD98059, LY294002 and AG490, respectively (Fig. 7A-C).

### DR1-CSE/H_2_S pathway activation inhibits proliferation by down-regulating IGF-1/IGF-1R and HB-EGF/EGFR

DR1 was overexpressed, CSE, IGF-1 and HB-EGF were knocked down in HG-induced MOVAS. We found that the transfection efficiency of DR1, CSE, IGF-1 and HB-EGF was 40-60% (Supplementary Fig. 2A-D). Overexpression of DR1 obviously decreased the expression of PCNA and Cyclin D1 and increased the expression of p21 (compared with the control vector + HG group). The knock-down of CSE corresponded to the effect of PPG and reversed the effect of SKF38393 in inhibiting proliferation. The result was an increase in the expression of PCNA and CyclinD1 and a decrease in the expression of p21 (compared with the over DR1 + HG group). The knock-down of IGF-1 and HB-EGF also down-regulated the proliferation effect induced by HG, thus decreasing the expression of PCNA and CyclinD1 increasing the expression of p21. The effect of overexpression of DR1 was similar to the knock-down of IGF-1 and HB-EGF (Fig. 7D-I).

### DR1 activates CaM by up-regulating intracellular calcium, thereby activating the CSE/H_2_S pathway in HG-induced MOVAS

**Figure 8.**
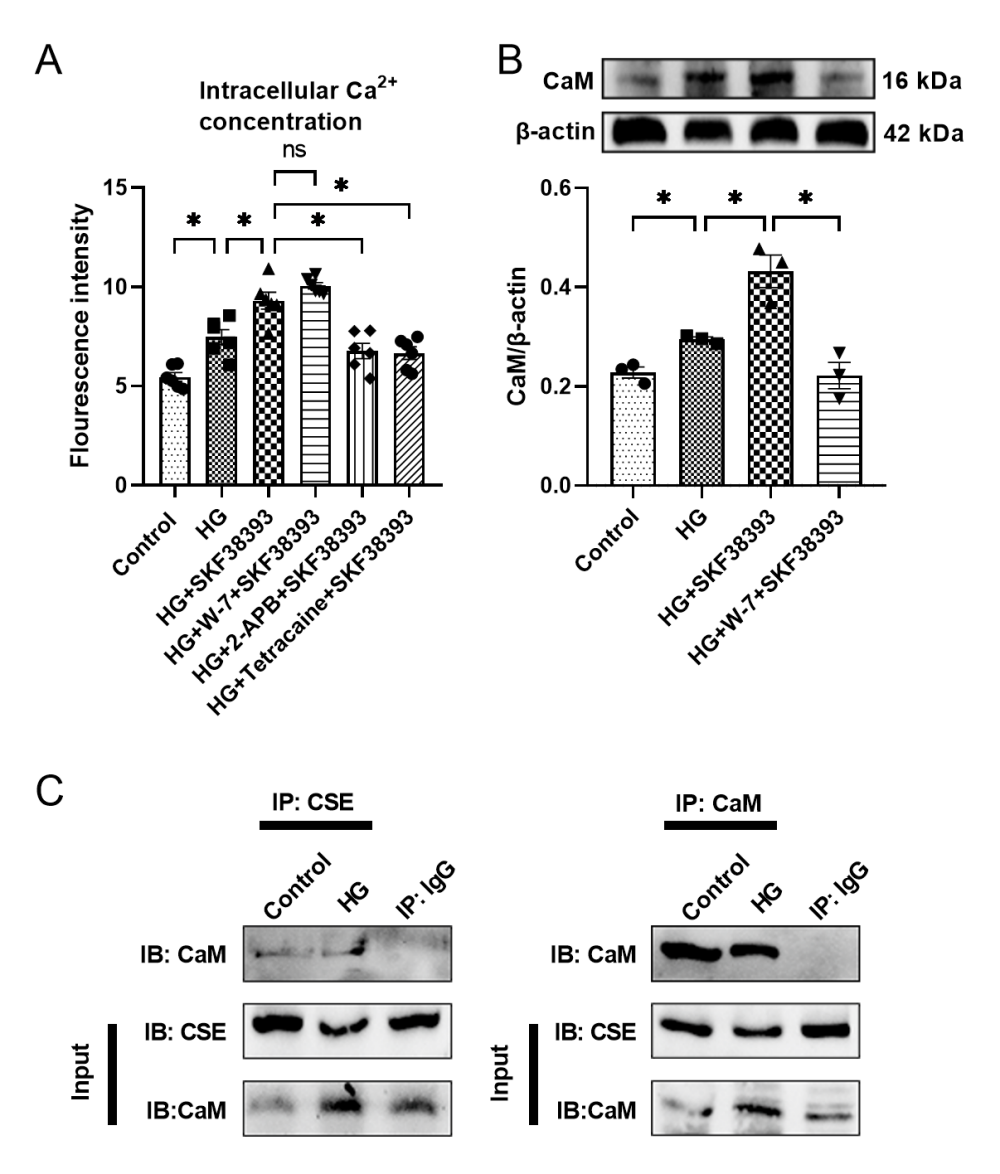
DR1 activates CaM by up-regulating intracellular calcium and CaM directly combined with CSE. (A) Determination of intracellular calcium levels by Flow-4 ion probe by fluorescence enzyme-labeled instrument (n=6). Western blot analysis observed the expression of CaM (B) in different cell experiment groups (n=3). The intensity of each band was quantified by densitometry, and data were normalized to the β-actin signal. (C) Co-immunoprecipitation was used to detect the interaction of CaM and CSE. The results were expressed as the mean ± SEM. Significant differences are indicated as * p < 0.05. ns, not significant.

In order to explore the related mechanisms of DR1 activating the CSE/H_2_S pathway, [Ca^2+^]_i_, the expression of CaM and the interaction of DR1 and CSE were detected in HG-induced MOVAS. The [Ca^2+^]_i_ of the HG group was higher than the control group. After SKF38393 was given, the [Ca^2+^]_i_ was further up-regulated. The addition of W-7, an inhibitor of CaM, did not affect the up-regulation effect of HG and SKF38393 on the [Ca^2+^]_i_. 2-APB (an IP_3_ inhibitor) and tetracaine (a RyR inhibitor) closed the channel for internal calcium release and reduced the increase in intracellular calcium caused by SKF38393 (Fig. 8A). Up-regulation of the [Ca^2+^]_i_ will inevitably cause the activation of CaM. The expression of CaM was tested in subsequent experiments. Under HG culture, the expression of CaM was higher than in the control group and further increased in the HG+SKF38393 group. CaM inhibitor W-7 abolished the effect of SKF38393 (Fig. 8B). Finally, it was proven that CaM and CSE can interact directly in the cell through co-immunoprecipitation experiments (Fig. 8C). These results suggest that DR1 activation up-regulates the CSE/H_2_S pathway by increasing the activity of the Ca^2+^-CaM pathway.

## DISCUSSION

Our experiment proved for the first time that DR1 activates the CSE/H_2_S pathway and releases endogenous H_2_S by increasing [Ca^2+^]_i_ to activate CaM, thereby inhibiting the proliferation of VSMCs. The mechanism of H_2_S inhibiting VSMCs is the activation of IGF-1/IGF-1R and HB-EGF/EGFR as well as their downstream PI3K/Akt, JAK2/STAT3 and ERK1/2 pathways.

Diabetes mellitus (DM) is one of the most common metabolic disorders and is estimated to affect 4.4% of the global population in the next 20 years [29]. The vast majority of diabetes is divided into two categories, type 1 diabetes: an absolute lack of insulin secretion, and type 2 diabetes: the cause is a combination of resistance to insulin action and an inadequate compensatory insulin secretory response [30]. Due to lack of insulin or insulin resistance, the extracellular glucose concentration increases significantly [2]. The elevated blood glucose is a potential cause of cardiovascular disease [31]. Cardiovascular complications in diabetic patients have a high lethality rate [32], which may be due to the proliferation and migration of VSMCs under high glucose conditions [33]. The abnormal proliferation and migration of VSMCs is an important pathophysiological process that accelerates diabetic vascular complications [34]. Therefore, inhibiting the abnormal proliferation and migration of VSMCs is of great significance for the prevention and treatment of diabetes mellitus and its vascular complications [35]. Based on this background, we used STZ to induce mice to become a type 1 diabetes model to simulate hyperglycemia [36] and achieve the purpose of inducing the proliferation of mouse aortic SMCs. Experiments have shown that the level of dopamine in the striatum of diabetic mice is reduced [37] and the gene expression of DR1 in diabetic rats is reduced [38]. Our experimental results also proved that the proliferation of VSMCs was increased and the expression of DR1 and CSE and H_2_S production were decreased in the arteries of T1DM mice and HG-induced VSMCs. This indicates that HG-induced VSMCs proliferation is related to decreased DR1 expression and the generation of endogenous H_2_S (Fig. 1B-E).

**Figure 9.**
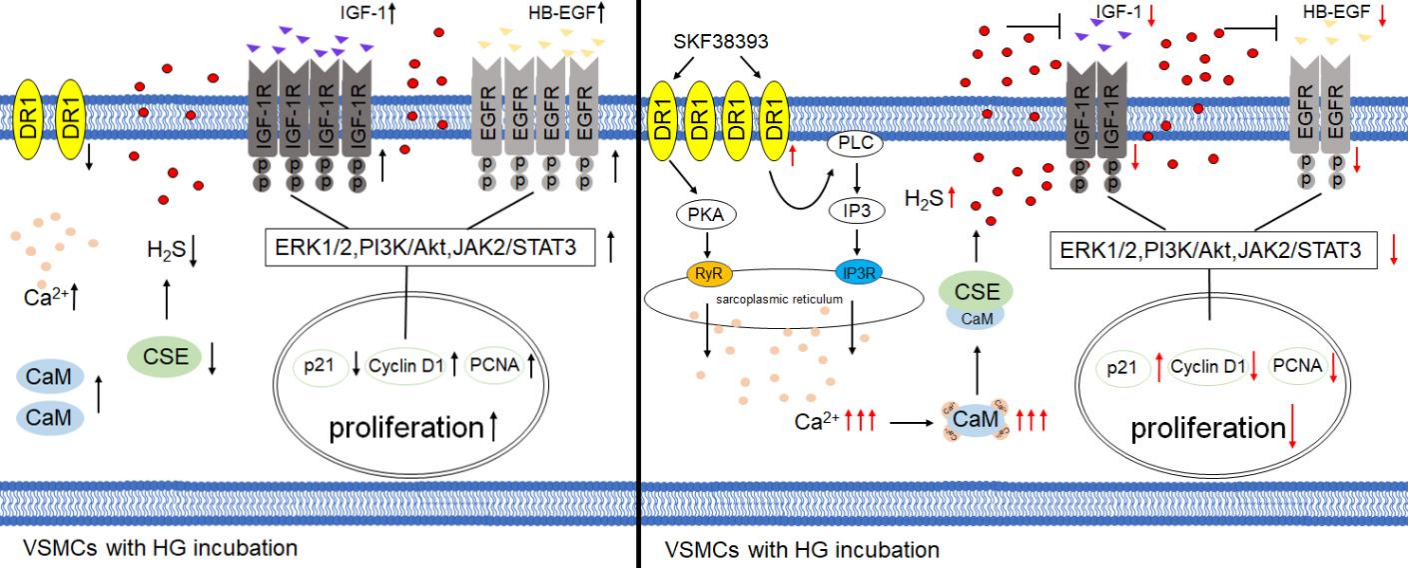
Conclusion diagram. In the case of HG, the expression of DR1 and CSE/H_2_S system are decreased, IGF-1/IGF-1R and HB-EGF/EGFR as well as their downstream PI3K/Akt, JAK2/STAT3 and ERK1/2 pathways are activated, VSMCs are in a state of abnormal proliferation. DR1 activation via DR1 agonist SKF38393 up-regulates CSE/H_2_S pathway by increasing Ca^2+^- CaM combination, which down-regulates IGF-1/IGF-1R and HB-EGF/EGFR pathways, thereby decreasing its downstream PI3K/Akt, JAK2/STAT3 and ERK1/2 pathways to achieve the effect of inhibiting HG-induced VSMCs proliferation.

To further confirm this viewpoint and the interrelation of DR1 and endogenous H_2_S, we supplemented with a DR1 agonist SKF38393, an exogenous H_2_S donor NaHS and a CSE inhibitor PPG in vivo and in vitro and found that SKF38393 significantly increased DR1 and CSE expression and endogenous H_2_S production, while NaHS only increased CSE expression and endogenous H_2_S production, but had no effect on DR1 expression. The CSE inhibitor PPG abolished the effect of SKF38393 (Fig. 2 and 4). These results suggest that DR1 activation inhibits HG-induced VSMCs proliferation by increasing endogenous H_2_S production. DR1 is upstream regulatory factor of CSE/H_2_S system.

In recent years, H_2_S has been shown to inhibit the proliferation of VSMCs under HG conditions [19]. H_2_S inhibits VSMCs proliferation by down-regulating Cyclin D1 and up-regulating p21^Cip/WAF-1^ pathways, thus blocking the cell cycle in the G1 phase [39]. At the same time, H_2_S can also reduce the expression of PCNA to achieve an effect of inhibiting the proliferation of VSMCs [40]. As an important member of the cyclin-dependent kinase inhibitor family, p21 can not only inhibit the expression of cyclin-dependent kinases (CDKs) [41] but also regulate the expression of PCNA. Blockage of the cell cycle depends on maintaining a high concentration of p21^Cip/WAF-1^, which in turn reduces the level of PCNA [42]. The data of in vivo and in vitro experiments showed that HG significantly increased cell viability and proliferation and the expression of PCNA and Cyclin D1 and reduced the expression of p21. SKF38383 and NaHS reversed the effect of HG. PPG blocked the effect of SKF38393 on cell proliferation (Fig. 3 and 5). Taken together, our results demonstrate that DR1 activation increases endogenous H_2_S production, which inhibits HG-induced VSMCs proliferation by affecting the expression of cell cycle-associated proteins.

Insulin-like growth factor-I (IGF-1) is a pleiotropic hormone that regulates VSMCs migration, proliferation, apoptosis, and differentiation. These actions are mediated by IGF-1R [43]. Rui Wang et al. recently showed that IGF-1 stimulated the proliferation of SMCs by binding to IGF-1Rs [39]. Both endogenous and exogenous H_2_S down-regulated the expression of IGF-1R and induced IGF-1R S-sulfhydration in aortic tissues or aortic SMCs [44]. Heparin-binding EGF-like growth factor (HB-EGF) is a member of the EGF family, which binds to and activates EGFRs, and is mainly manifested in various tissues such as the lung, heart, brain and skeletal muscle [45, 46]. The activation of EGFR promotes VSMCs proliferation, migration, inflammation and fibrosis, and participates in the process of vascular remodeling in vascular diseases (such as hypertension and atherosclerosis) [47]. IGF-1R signaling activates multiple downstream signaling pathways, including PI3-kinase, Akt, and mitogen-activated protein kinase (MAPK) [48]. EGFR induces VSMCs proliferation by activating the PI3K/Akt and MAPK pathways [45]. The JAK2/STAT3 pathway can regulate gene expression in various biological processes, including proliferation, cell survival and inflammation [49]. Phosphorylation of STAT3 induced trans-activation of Cyclin D1 in SMCs in vitro and in neointimal cells in vivo, thus promoting the proliferation and migration of SMCs as well as reducing apoptotic cell death [50]. Our results showed HG enhanced the pathways of IGF-1/IGF-1R, HB-EGF/EGFR, PI3K/Akt, JAK2/STAT3 and ERK1/2 and the expression of PCNA and Cyclin D1 and reduced the expression of p21. SKF38393 and NaHS markedly reversed the HG-induced changes. PPG abolished the effect of SKF38393. The effect of SKF38393 was similar to the respective inhibitors of the corresponding pathways. Meanwhile, both IGF-1 inhibitor AG1024 and HB-EGF inhibitor AG1478 also suppressed the PI3K/Akt, JAK2/STAT3 and ERK1/2 pathways (Fig. 6 and 7A-C). In addition, we also found that the overexpression of DR1 decreased the expression of PCNA and Cyclin D1 and increased the expression of p21. Knock-down of CSE reversed the effect of DR1 activation on proliferation. The effect of overexpression DR1 on proliferation related factors was similar to knocking out IGF-1 and HB-EGF, respectively (Fig. 7D-I). These findings suggest that DR1 activation increases endogenous H_2_S production, which inhibits HG-induced VSMCs proliferation by down-regulating IGF-1/IGF-1R and HB-EGF/EGFR, as well as their downstream PI3K/Akt, JAK2/STAT3 and ERK1/2 pathways.

The above experimental results confirmed that DR1 activation inhibits VSMCs proliferation by increasing endogenous H_2_S production in T1MD mice and HG-induced VSMCs. How does the activation of DR1 increase the production of endogenous H_2_S? To answer this question, we tested [Ca^2+^]_i_, the expression of CaM and the interaction of CaM and CSE. In the case of HG, the level of [Ca^2+^]_i_ is increased (Fig. 8A). This is similar to the results of the previously published literature. The HG concentration leads to an increase in [Ca^2+^]_i_ in a dose- and time-dependent manner without other stimuli [51, 52]. The activation of IP3 and RyR receptors is the main reason for the increase in [Ca^2+^]_i_[53, 54]. In addition, in VSMCs, the IGF-1/IGF-1R and EGF/EGFR pathways activate Ca^2+^ channels such as the TRP classical (TRPC) type 3 and 6 and TRPM4 channels, and voltage-dependent calcium channels, thereby up-regulating Ca^2+^ regulated myogenic tone [47]. Ca^2+^ is a crucial regulatory ligand, and among its many binding partners, CaM serves as its primary intracellular receptor for controlling essentially every aspect of cellular biology [55]. A direct relationship between an increase in the levels of intracellular free Ca^2+^ and the Ca^2+^-dependent activation of CaM was proven earlier [56]. Yang et al. found that Ca^2+^-CaM pathway activation up-regulated CSE/H_2_S system in the vascular endothelial cells [22]. Other researchers also reported that activation of the Ca^2+^-CaM pathway inhibited the proliferation of VSMCs by enhancing the CSE/H_2_S pathway [57]. Our previous results demonstrated that DR1 activation promoted myocardial ischemia-reperfusion injury by increasing[Ca^2+^]_i_ [23]. Our data showed that CaM interacts with CSE (Fig. 8C). Meanwhile, HG increased [Ca^2+^]_i_. SKF38393 further increased [Ca^2+^]_i_. 2-APB (an IP3R inhibitor) and tetracaine (a RyR receptor inhibitor) blocked the effect of SKF38393 (Fig. 8A). In addition, HG increased the expression of CaM. SKF38393 further increased CaM expression. W-7 abolished the effect of SKF38393 (Fig. 8B). These results suggest that DR1 activation increases endogenous H_2_S production by enhancing Ca^2+^-CaM pathway in HG-induced VSMCs.

In summary (Fig. 9), in the case of HG, the expression of DR1 and CSE/H_2_S system are decreased, IGF-1/IGF-1R and HB-EGF/EGFR as well as their downstream PI3K/Akt, JAK2/STAT3 and ERK1/2 pathways are activated, VSMCs are in a state of abnormal proliferation. Administration of DR1 agonists SKF38393 can increase [Ca^2+^]_i_ and activate CaM, thereby activating the CSE/H_2_S system, releasing endogenous H_2_S, and then down-regulating IGF-1/IGF-1R and HB-EGF/EGFR as well as their downstream PI3K/Akt, JAK2/STAT3 and ERK1/2 pathways, finally, inhibiting VSMCs proliferation. A potential target was found for the up-regulation of H_2_S, so as to achieve the protective effect of H_2_S on diabetes mellitus and its complications.

## Data and materials availability

The data set used and/or analyzed in the current research can be obtained from the corresponding author upon reasonable request.

## Supplementary Materials

The Supplementary data can be found online at: www.aginganddisease.org/EN/10.14336/AD.2021.1104.


