# Supplementary material for: DR1 Activation Inhibits the Proliferation of Vascular Smooth Muscle Cells through Increasing Endogenous H2S in Diabetes
Source: Aging Dis. 2022 Jun 1;13(3):910–26. doi: 10.14336/AD.2021.1104 (PMC9116912; doi:10.14336/AD.2021.1104)
Supplement: Supplementary file 1 [file AD-13-3-910-s.pdf]

SUPPLEMENTARY DATA

DR1 Activation Inhibits the Proliferation of Vascular Smooth Muscle Cells through Increasing Endogenous H_2S in Diabetes

Yuxin Xi[1,#], Xin Wen[1,#], Yuanzhou Zhang[3], Lijie Jiao[2], Shuzhi Bai[1], Sa Shi[1], Guiquan Chang[1], Ren Wu[1], Fengqi Sun[1], Jinghui Hao[1], Hongzhu Li[1,2,*]

© 2021. Xi Y et al. Published online at http://www.aginganddisease.org/EN/10.14336/AD.2021.1104

SUPPLEMENTARY DATA

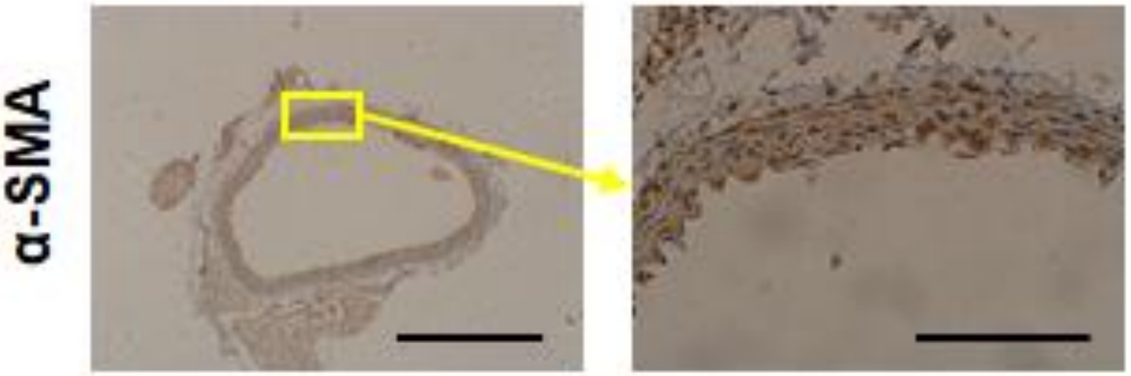

Supplementary Figure 1. Identification of aortic smooth muscle cells (SMCs). The expression of α-SMA was observed by immunohistochemical method in the normal aortic SMCs of mice. The expression of α-actin is brown (100× or 600 × magnification). Scale bar in aortic ring: 500 μm; Scale bar in part of aorta: 100 μm.

Supplementary Table 1. Blood glucose, weight, water intake and food intake of diabetic mice in 4w, 8w and 12w (n=8).

	Blood Glucose (mmol/L)	Weight (g)	Water Intake (ml/d)	Food Intake (g/d)
Control 4w	3.8 ± 0.5	19.0 ± 1.6	3.4 ± 0.6	2.7 ± 0.4
T1DM 4w	$21.8\pm5.8^{*}$	18.0 ± 2.2	$20.5\pm3.1^{*}$	$3.9\pm0.4^{*}$
Control 8w	3.9 ± 0.4	19.8 ± 2.2	3.4 ± 0.4	3.1 ± 0.1
T1DM 8w	$24.0\pm6.3^{\#}$	18.0 ± 2.1	$20.7\pm1.5^{\#}$	$4.0\pm0.2^{\#}$
Control 12w	3.9 ± 0.7	20.0 ± 1.5	3.7 ± 0.2	3.4 ± 0.1
T1DM 12w	$24.6\pm5.7^{\&}$	18.2 ± 1.7	$23.2\pm1.4^{\&}$	$4.5\pm0.2^{\&}$

T1DM: type 1 diabetes mellitus; * $p < 0.05$ vs. the control 4w group; # $p < 0.05$ vs. the control 8w group; & $p < 0.05$ vs. the control 12w group.

© 2021. Xi Y et al. Published online at http://www.aginganddisease.org/EN/10.14336/AD.2021.1104

SUPPLEMENTARY DATA

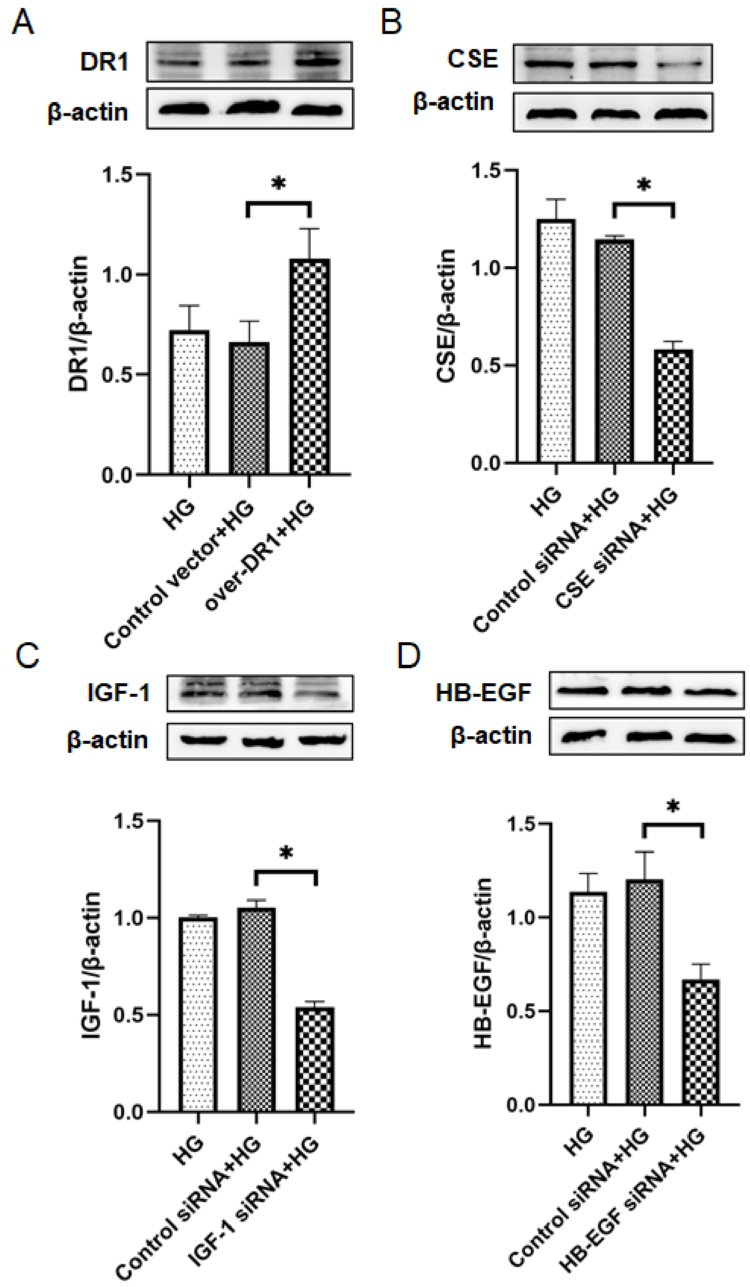

Supplementary Figure 2. The transfect efficiency of over-DR1 (A), CSE siRNA (B), IGF-1 siRNA (C) and HB-EGF siRNA (D). The transfect efficiency of over-DR1, CSE siRNA, IGF-1 siRNA and HB-EGF siRNA was detected by Western blot. Over-DR1 is the overexpression of DR1 gene, CSE siRNA is knock down CSE gene. IGF-1 siRNA is knock down IGF-1 gene. HB-EGF siRNA is knock out HB-EGF gene. Transfection efficiency is 40-60%. All data were from three independent experiments. The results were expressed as the mean±SEM. Significant differences are indicated as * $p < 0.05$.

© 2021. Xi Y et al. Published online at http://www.aginganddisease.org/EN/10.14336/AD.2021.1104
